# *Clostridium perfringens* Epsilon Toxin: A Malevolent Molecule for Animals and Man?

**DOI:** 10.3390/toxins5112138

**Published:** 2013-11-12

**Authors:** Bradley G. Stiles, Gillian Barth, Holger Barth, Michel R. Popoff

**Affiliations:** 1 Biology Department, Wilson College, 1015 Philadelphia Avenue, Chambersburg, PA 17201, USA; 2 Veterinary Medical Technology Department, Wilson College, 1015 Philadelphia Avenue, Chambersburg, PA 17201, USA; E-Mail: gillian.barth@wilson.edu; 3 Institute of Pharmacology and Toxicology, University of Ulm Medical Center, Albert-Einstein-Allee 11, Ulm D-89081, Germany; E-Mail: holger.barth@uni-ulm.de; 4 Bacteries Anaerobies et Toxines, Institut Pasteur, 28 rue du Docteur Roux, Paris 75724, France; E-Mail: mpopoff@pasteur.fr

**Keywords:** *Clostridium perfringens*, protein toxin, receptor, animal models, cell culture, vaccines, therapeutics

## Abstract

*Clostridium perfringens* is a prolific, toxin-producing anaerobe causing multiple diseases in humans and animals. One of these toxins is epsilon, a 33 kDa protein produced by *Clostridium perfringens* (types B and D) that induces fatal enteric disease of goats, sheep and cattle. Epsilon toxin (Etx) belongs to the aerolysin-like toxin family. It contains three distinct domains, is proteolytically-activated and forms oligomeric pores on cell surfaces via a lipid raft-associated protein(s). Vaccination controls Etx-induced disease in the field. However, therapeutic measures are currently lacking. This review initially introduces *C. perfringens* toxins, subsequently focusing upon the Etx and its biochemistry, disease characteristics in various animals that include laboratory models (*in vitro* and *in vivo*), and finally control mechanisms (vaccines and therapeutics).

## 1. Introduction

*Clostridium perfringens* is a Gram-positive, spore-forming anaerobe residing in soil, water as well as the gastrointestinal tracts of various mammals, including humans. This ubiquitous bacillus is one of the most “toxic” of all known bacteria, collectively producing more than 15 different protein toxins/enzymes with diverse modes of action [1,2]. Pathogenic *Clostridium* species synthesize some of the most potent toxins that include tetanus and botulinum neurotoxins, respectively produced by *Clostridium tetani* and *Clostridium botulinum*. These bacteria are found in similar environments (*i.e*., soil) as long-lasting, quiescent spores that await a mammalian host and infection/intoxication opportunity. 

*C. perfringens* was first isolated by William Welch and George Nuttall (1892) at Johns Hopkins Hospital (Baltimore, MD, USA) following autopsy of a cancer/tuberculosis patient, eight hours post-death. In particular, they noted flammable gas bubbles perfused throughout the cadaver and especially within blood vessels. Gas (carbon dioxide plus hydrogen) and organic acids (acetic, butyric, lactic, *etc*.) are common byproducts of anaerobic metabolism by *C. perfringens. C. perfringens* has also been successively known in the literature as *Bacillus aerogenes capsulatus*, *Bacillus welchii* or *Clostridium welchii* [1,2,3]. Various diseases of animals and humans caused by *C. perfringens* are linked to protein toxins, and the next section succinctly describes the “major” and “minor” toxins produced by this bacterium. One of those toxins, epsilon, is this review’s focus as this protein impacts in many ways the veterinary and biodefense fields throughout the world. 

## 2. *Clostridium perfringens* Toxins: Major and Minor (A Brief Overview)

Protein toxins are important virulence factors of *C. perfringens* and have been a research focus of various laboratories around the world. For bacterial pathogens, toxins possessing diverse modes of action often play critical roles during disease, including food gathering and suppressing the host’s immune system. The four major toxins produced by *C. perfringens* either affect cell membranes directly by increasing permeability and causing ion imbalances (alpha, beta and epsilon toxins), or destroy the actin cytoskeleton (iota toxin) [1,2]. Intoxication by any of these clostridial proteins ultimately leads to cell dysfunction and death, as well as host suffering that can become fatal. Like the spores formed by other *Clostridium* and *Bacillus* species that enable survival in soil, protein toxins can play a pivotal role in bacteria surviving and subsequently thriving in an animal or human host. 

There are five toxin types (A, B, C, D and E) of *C. perfringens* based upon the production of one or more major protein toxins [1,2] (Table 1). These toxins are linked to diverse diseases/intoxications of humans and/or animals (Table 2). 

**Table 1:** Major toxins for *C. perfringens* typing.

Toxin	*C. perfringens* Type					Cellular Target (mode of action)
	A	B	C	D	E	
Alpha	+	+	+	+	+	Membrane (phospholipid destruction)
Beta		+	+			Membrane (pore formation)
Epsilon		+		+		Membrane (pore formation)
Iota					+	Actin (cytoskeleton destruction)

**Table 2:** *C. perfringens* toxin types and associated diseases.

Toxin Type	Disease
A	Myonecrosis (gas gangrene in humans and animals); Necrotic enteritis of fowl plus piglets; Human food poisoning and antibiotic-associated diarrhea
B	Hemorrhagic enteritis in calves, foals and sheep; Dysentery in lambs
C	Necrotizing enteritis in humans (also popularly called pigbel, darmbrand or fire-belly), as well as in pigs, calves, goats and foals; Enterotoxemia in sheep (alias struck)
D	Enterotoxemia in lambs (known as pulpy kidney disease), goats and cattle
E	Enterotoxemia in calves and lambs. Similar enteric disease induced by iota-like toxin in rabbits, caused by *Clostridium spiroforme*

For diagnostic purposes, major toxins of *C. perfringens* found in field samples or cultured isolates *in vitro* were historically neutralized in the laboratory by type-specific antisera in mouse-lethal and guinea-pig dermonecrotic assays [3]. Rapid genetic methods employing multiplex polymerase chain reaction (PCR) are now much more common for typing *C. perfringens* [4,5]. This technique is accurate and rapid; however, PCR merely suggests a gene’s presence and indicates neither expression levels nor quantities of an effector molecule (biologically-active toxin) that are ultimately responsible for causing physiological changes to a cell. 

Detection of all major *C. perfringens* toxins has also been reported by various laboratories using ELISA technology [6,7,8,9]. Quantitation of epsilon toxin protein is also possible using a novel, mass spectrometry technique [10]. In contrast to ELISA or mass spectrometry, animal assays and toxin-susceptible cell cultures can effectively determine if biologically-active toxin (in conjunction with toxin-specific antibody use) exists in a sample. For any biological protein in a suspect sample, structural integrity is linked to many factors that include how quickly the sample is collected post-mortem, room temperature *vs.* refrigerated/frozen storage, how long the sample is stored before testing, *etc*. In particular, enterotoxins found in digesta can be altered by many “factors” such as proteases and non-specific protein binding naturally found in the intestinal tract. 

### 2.1. Alpha Toxin

Type A strains of *C. perfringens* are most commonly found throughout the environment and linked to gas gangrene of animals and humans [1,11,12,13,14,15]. Alpha toxin facilitates gas gangrene due to *C. perfringens* infection, a life-threatening myonecrotic disease historically common with battlefield wounds [11,12]. Deep, penetrative wounds contaminated by soil harboring various clostridial species, including *C. perfringens*, are often to blame for this quickly advancing disease of thick-muscled body regions that include the buttocks, shoulder, arm and leg [11,14,15]. Prior to the 20th century and a true understanding of antisepsis, with a knowledge of specific etiological agents that cause infectious diseases like gangrene, an extremity wound could quickly result in an amputation that simply would not occur with today’s modern medicine [15]. Rapid treatment involving extensive surgical debridement, various antibiotics that include beta-lactams, clindamycin and/or metronidazole, as well as hyperbaric oxygen prove effective for most cases of *C. perfringens*-induced gangrene. Anti-toxin (historically, serum antibodies of equine origin) is another possible therapy for mitigating alpha-toxin induced myonecrosis [11,14,16]. Recombinant-based technology generating human monoclonal antibodies could replace equine polyclonal, circumventing potentially life-threatening serum sickness. Regarding a prophylaxis perspective, vaccine studies from various groups using the carboxy-terminal (cell binding) domain of alpha toxin show protection in mice against either toxin-induced lethality or bacterial challenge in a gangrene model [17,18]. 

Alpha toxin is a zinc-containing phospholipase C (43 kDa), composed of two structural domains, that destroys eukaryotic cell membranes [19,20]. In 1941, *C. perfringens* alpha was the first bacterial toxin ascribed enzymatic activity [21]. Like beta, but unlike epsilon and iota, the alpha toxin is relatively susceptible to proteolysis by serine-type proteases such as trypsin and chymotrypsin. The amino-terminal domain contains a catalytic site and ganglioside (GM1a) binding motif, the latter being curiously similar to that found on *C. botulinum* neurotoxin [22]. Interaction of GM1a with alpha toxin promotes clustering/activation of tyrosine kinase A involved in signal transduction. The carboxy-terminal domain of alpha toxin binds to membrane phospholipids.

### 2.2. Beta Toxin

Originally purified in 1977, beta toxin is a 35 kDa protein that shares sequence similarity with the alpha and gamma hemolysins of *Staphylococcus aureus* [23,24]. The toxin is responsible for fatal necrotic enteritis in animals and humans involving intestinal necrosis and bloody stools. In humans, diseases such as pigbel (Papua, New Guinea) or Darmbrand (post-World War II Germany) follow consumption of meat by individuals on a minimal protein diet with a low-basal level of pancreatic trypsin [25]. For some pigbel cases, individuals may have consumed trypsin inhibitor via sweet potatoes (a staple component of the normal diet) and/or be infected by round worms (*Ascaris lubricoides*) that release trypsin inhibitor into the intestinal lumen. Unusually high concentrations of protein in the intestinal tract facilitate *C. perfringens* types B (animal) or C (human) overgrowth, leading to lethal levels of beta toxin, which forms cation-selective channels in lipid membranes [26]. Tachykinin (neuropeptide) receptors play a role in beta-toxin induced fluid release from the circulatory system into tissue, suggesting involvement of the sensory nervous system [27]. This particular study in murine dermis reveals that beta intoxication is inhibited by tachykinin NK_1_ antagonists, capsaicin and an omega conotoxin (*Conus magus* MVIIA) that specifically blocks N-type calcium-channels.

### 2.3. Iota Toxin

Iota toxin, discovered in 1943 by Bosworth [28], is unique among the major toxins of *C. perfringens* by consisting of two non-linked proteins that form a multimeric complex on susceptible cells, akin to the edema and lethal binary toxins from *Bacillus anthracis* [29]. Like *C. perfringens*, *B. anthracis* is a Gram-positive sporulating bacillus found in soil that uniquely causes various forms of anthrax (dermal, enteric and pneumonic) in animals as well as humans. For iota toxin, the cell-binding component (Ib or iota b) is an 81 kDa monomer that forms a heptamer following proteolytic activation of the 94 kDa protoxin. The complementary enzymatic component is an ADP-ribosyl transferase (Ia or iota a) of 45 kDa, which in complex with the Ib heptamer, travels from the cell surface via lipid rafts into endosomes. The Ia component translocates from an acidified endosome into the cytosol, through a transmembrane pore formed by the Ib heptamers. In the cytosol, Ia covalently transfers an ADP-ribose moiety from nicotinamide adenine dinucleotide (NAD) to Arg177 on globular (G) actin, which then prevents filamentous (F) actin formation. Destruction of the cytoskeleton ensues, ultimately killing an intoxicated cell unable to maintain intracellular trafficking necessary for homeostasis.

In addition to the major toxins listed above, there is a sporulation-linked *C. perfringens* enterotoxin that causes a major form of food poisoning linked to meat consumption [30]. Finally, there are other *C. perfringens* proteins designated as minor toxins that can play prominent roles in pathogenesis. A list of these latter toxins/enzymes with putative activities is presented in Table 3. 

**Table 3:** Minor toxins/enzymes of *C. perfringens*.

Toxin/Enzyme	Activity
Beta 2	?
Delta	Cytolysin
Eta	?
Gamma	?
Kappa	Collagenase
Lambda	Protease
Mu	Hyaluronidase
Nu	Deoxyribonuclease
NanI, NanJ, NanH	Neuraminidase
NetB	Hemolysin
Theta (perfringolysin O)	Oxygen-labile Hemolysin
TpeL	Glucosylation of Ras

## 3. Epsilon Toxin (Etx): *C. perfringens* Most Toxic Toxin

### 3.1. Natural Occurrence and a Potential Biological Warfare/Terrorism Agent?

Etx produced by *C. perfringens* types B and D is involved in animal (goats, sheep and less frequently cattle) enterotoxemias that can be rapidly fatal and economically devastating [1,2,31]. Etx is the most potent of all *C. perfringens* toxins as determined by a 50% lethal dose (LD_50_ of ~70 ng/kg body weight, ranking behind only the *C. botulinum* and *C. tetani* neurotoxins in classic mouse-lethal assays commonly used for clostridial toxins. *C. perfringens* is considered normal intestinal flora in ruminants. Resident types B and D can cause life-threatening illness in a “naïve” digestive system shortly after birth or following a diet change involving more carbohydrates. Given proper conditions, *C. perfringens* can rapidly proliferate in the intestines and concomitantly produce life-threatening levels of toxins, including Etx. The disease is strictly a toxemia that spills into the circulatory system, since bacterial invasion of intestinal tissue is not common. Experts naturally associate the Etx with veterinary maladies. Unlike various studies from multiple groups involving different animal species, the existing literature simply does not suggest that human intoxication by Etx naturally occurs even infrequently. 

Due to national and international concerns involving biological warfare/terrorism, *C. perfringens* Etx has thus received much attention from various governments [32]. The potential nefarious use of Etx against humans by rogues provides chilling insight into human psychology and fear mongering. The United States Department of Agriculture and Centers for Disease Control and Prevention have classified Etx as a select agent, like some bacterial diseases (brucellosis, glanders and typhus) plus other protein toxins such as ricin and staphylococcal enterotoxin B (SEB). However, modification in December 2012 of the select agents list has now removed *C. perfringens* Etx [33]. In France, Etx is still classified as a potential biological weapon requiring special authorization for laboratory work from the Agence Nationale de Securite du Medicament (ANSM). Varying opinions clearly exist around the world regarding *C. perfringens* Etx, its potential nefarious use to promote societal fear, and subsequently imposed oversight to protect the governed masses.

### 3.2. Chemical and Physical Properties

*C. perfringens* Etx is coded on a plasmid and secreted as a protoxin (32.9 kDa), subsequently activated by extracellular serine-type proteases (trypsin/chymotrypsin) that remove 10–13 amino- and 22 or 29 carboxy-terminal residues, depending on the protease [31]. The protoxin contains a typical leader sequence (32 residues) that facilitates secretion from the bacterium’s cytosol into the environment. Activated toxin (29 kDa) is relatively resistant to proteases in the gastrointestinal tracts of mammals. 

**Figure 1:**
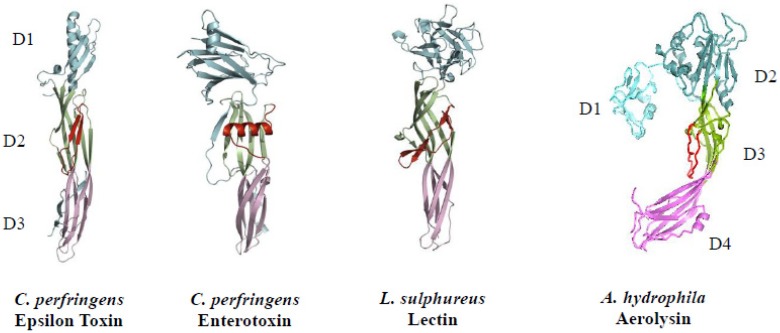
Crystal structures of *C. perfringens* epsilon toxin, *C. perfringens* enterotoxin, *L. sulphureus* lectin and *A. hydrophila* aerolysin. A basic, three-domain structure is evident for each protein [30,34,35,36,37]. Amino acids involved in pore-formation are in red.

Etx is an elongated, beta-sheet (100 Å × 20 Å × 20 Å) composed of three domains sharing conformation with other bacterial pore-forming toxins (PFTs) of the aerolysin-like family, such as *Aeromonas hydrophila* aerolysin and *C. perfringens* enterotoxin (Figure 1) [30,34,35]. 

Furthermore, this protein family is produced by other diverse life forms that include a Brazilian tree (*Enterolobium contortisiliquum*), mushroom (*Laetiporus sulphureus*) and freshwater hydra (*Chlorohydra viridis*) [35]. Although primary sequence homology among the aerolysin-like family is relatively low (< 20% identity), conformations are strikingly similar. A hallmark of these convergently-evolving proteins involves synthesis as monomers that generate homo-oligomeric (hexameric or heptameric) complexes on a cell-surface membrane, leading to transmembrane pore formation.

Two groups of PFTs exist, based upon using either amphipathic alpha-helix (alpha-PFT) or beta-hairpin (beta-PFT) structures for membrane insertion. Etx is a beta-PFT. Putative roles for Etx domains include: receptor binding (domain I–amino terminus); membrane insertion plus channel formation (domain II–central region); and proteolysis activation plus monomer-monomer interaction sites (domain III–carboxy terminus) [34,35]. Following loss of a carboxy-terminal peptide from epsilon protoxin, there are subsequent monomer-monomer interactions that lead to homo-heptamer formation [38]. 

Proteolysis is a common process that activates many bacterial toxins. For epsilon, this process induces conformational changes that facilitate homo-oligomerization of activated toxin on the external surface of a targeted, eukaryotic cell. This “protein priming” enables Etx to quickly act after binding to diverse target cells that include those of neuronal, renal and endothelial origins (described in detail below). Loss of the amino- and carboxy-termini from the epsilon protoxin generates a more acidic protein (isoelectric point of 5.4 *vs*. 8.0), perhaps favoring more productive receptor interactions [31,39]. For bacterial toxins produced intestinally and requiring proteolysis, proteases synthesized by resident bacteria (including *C. perfringens* lambda toxin, a 35 kDa thermolysin-like metalloenzyme) and the host are abundant [38,40]. Recent evidence, contrary to the existing paradigm, reveals that Etx can be activated intracellularly (in a select type D strain lacking lambda toxin), remains in *C. perfringens* until stationary/death phase and is then released into the environment after autolysis [41]. Identity of this protease, and a better understanding of this novel activation mode perhaps found in other protoxin-producing pathogens, remains a mystery. 

### 3.3. Cellular Target (Mode of Action)

Etx induces pore formation in eukaryotic cell membranes via detergent-resistant, cholesterol-rich membrane domains (lipid rafts) that promote aggregation of toxin monomers into homo-heptamers [42,43]. The pro- and activated-toxin forms bind to lipid rafts, but the former do not generate oligomers. Lipid rafts play important roles in many diseases caused by bacteria (as well as associated toxins) and viruses [44]. In particular, caveolins 1 and 2 found in these extracellular domains on ACHN (human adenocarcinoma kidney) cells bind to Etx and facilitate toxin oligomerization [45]. Caveolins (1–3) are a family of integral-membrane proteins (~20 kDa each) important in caveolae-mediated endocytosis and cell signaling. An Etx oligomer (155 kDa), critical for biological activity: (1) rapidly (within 15 minutes) forms at 37 °C on the surface of Madin-Darby canine kidney (MDCK) cells; (2) is more stable towards sodium dodecyl sulfate and heat (100 °C) when formed at 37 °C *vs*. 4 °C; and (3) becomes internalized from the cell surface, promoting vacuole formation in the late endosomes and lysosomes [46,47]. Epsilon-treated MDCK cells readily swell, form multiple blebs, and ultimately lyse; however, blocking of acid-mediated endocytosis via chloroquine, monensin, or bafilomycin A1 has no effect upon the intoxication process [46]. Pretreatment of cells with proteinase K results in a smaller (95 kDa) Etx complex, suggesting involvement of a surface protein(s) during toxin oligomerization [46]. 

Sialidases (neuraminidases) produced by *C. perfringens* enhance binding of the bacterium and Etx to cultured cells [48]. Interestingly, certain mammalian cell lines upregulate their sialidase genes upon contact with *C. perfringens*. Increased toxin binding following sialidase activity has been described previously for another enteric pathogen, *Vibrio cholerae*, perhaps suggesting a conserved pathogenic mechanism between diverse, enterotoxin-producing bacteria [49]. 

When applied to cells, Etx forms transmembrane pores (≥2 nm diameter) that rapidly facilitate: (1) free passage of 1 kDa-sized molecules; (2) decreased intracellular potassium levels; and (3) increased intracellular levels of chloride and sodium [46,50]. In addition to altering the membrane, secondary effects of epsilon intoxication involve cytoskeletal dysfunction [51] affecting integrity of cell monolayers [42]. Disruption of cell monolayers provides further understanding of the subsequent dysfunction of vascular endothelium, edema and crossing of the blood-brain barrier by the toxin as well as albumin-sized (~65 kDa) molecules [52,53]. 

## 4. A Veterinary Perspective on Etx: Field and Laboratory Findings

Sheep and goats are the most frequent, natural hosts for *C. perfringens* Etx, cattle less so [54,55]. *C. perfringens* type D strains are more common than type B for epsilon-induced disease, although both toxin types produce Etx. Various factors can play a role in disease, including: (1) consumption of feed rich in fermentable carbohydrates; (2) poor nutritional status; (3) parasite infestation; (4) pregnancy toxemia; (5) tranquilizer (phenothiazine) use; and (6) overdose of a broad spectrum anthelmintic (netobimin) [54,55]. Although Etx can be found in the heart, lungs, liver and stomach following intoxication, it noticeably accumulates in the kidneys, causing what veterinarians classically refer to as pulpy kidney, or overeating, disease [1,2,31,56,57,58]; however, evidence for pulpy kidney disease is inconsistent, probably a post-mortem effect and thus not considered diagnostic. A combination of toxin detected in the small intestines and brain lesions is a strong diagnostic. Another indicator that kidneys are a primary target of Etx is that the very few, susceptible cell lines discovered to date are mostly of kidney descent from dog, mouse and human [31].

Post-mortem findings in kidneys from either lambs or mice given Etx show similar results, including congestion, interstitial hemorrhage and degenerated epithelium in the distal tubules. Toxin accumulating in the kidney may be a host defense attempting to prevent lethal toxin concentrations in the brain [58]. During epsilon enterotoxemia of lambs, glucose excretion occurs via the urine and is perhaps a result of liver-released glycogen [59]. 

*C. perfringens* Etx rapidly disrupts the blood-brain barrier, binds neuronal cells and causes lethality [31,52,56,60]. Among neuronal cell populations, the neurons are most susceptible followed by oligodendrocytes and astrocytes [61]. There can be swelling, vacuolation and necrosis in the brain. Edema in the brain (rat) post-Etx exposure increases aquaporin-4 levels in astrocytes, which may be a defensive attempt to reduce osmotic pressure surrounding sensitive neurons [62]. Aquaporins (aquaglyceroporins) are a family (*n =* 13 members in mammals) of 30 kDa-sized proteins that form tetrameric, membrane-channel complexes regulating water flow in various cell types, perhaps representing an exploitable therapeutic target against Etx. There is already a keen medical interest in aquaporins as: (1) a diagnostic indicator for certain autoimmune diseases (*i.e*., Sjogren’s syndrome and nephrogenic diabetes insipidus); and (2) a therapy for glaucoma, meningitis, stroke, epilepsy, cancer, pain as well as weight regulation [63]. Cerebral swelling and necrosis of the brain following Etx exposure can be due to multiple factors like reduced blood flow, hypoxia and/or direct toxicity on various cell types within the brain. 

Clinical signs linked to Etx given intravenously to calves, lambs and kid goats are dose dependent and occur within minutes for calves and up to three hours in lambs [64,65]. For kids, either deprived or reared with colostrum by non-vaccinated dams, and given a 120, 185 or 250 mouse (M) LD_50_/kg body weight dose of Etx, clinical signs are evident in all within 95 minutes [64]. In this same study, weaned lambs (colostrum-reared from non-vaccinated dams) given a 120 or 250 MLD_50_/kg body weight dose of Etx show clinical signs within 170 minutes. These animals experience a myriad of symptoms involving labored breathing (*i.e*., foam-filled airways, alveolar edema, *etc*.), excited/exaggerated movements, intermittent convulsions, loss of consciousness and death. Additional signs of epsilon intoxication include elevated blood pressure, fluid in the lungs and brain congestion with edema [31,65]. There are remarkable histological differences between brain lesions in kids, calves and lambs. Perivascular leakage of protein is evident in lamb and calf, but not kid, brains; however, all of these animal types suffer from central nervous system distress post-Etx exposure. 

More natural than an intravenous injection of purified toxin, duodenal inoculation of Angora goats (12–14 kg kids) with either whole culture, culture supernatant or washed cells of *C. perfringens* type D leads to: (1) diarrhea (dark green and foul smelling containing bowel mucosa, fibrin and sometimes blood); (2) respiratory distress (lung edema and froth in trachea/bronchi); (3) glycosuria (although not a uniform response by all animals); and (4) central nervous system dysfunction (*i.e*., recumbency, bleating, convulsions and opisthotonos) [66]. Similar symptoms are also evident in lambs, minus diarrhea, pseudomembrane formation or any overt histological changes in the intestines [54,67]. In lambs there can be sudden death or neurological manifestations during acute disease that include struggling, opisthotonos, convulsions, lateral recumbency and violent paddling. 

Etx differentially affects sheep and goats, as the former have more overt brain effects (*i.e*., lesions) while the latter are more affected in the gut (*i.e*., diarrhea with structural damage to the colon, but not small intestine) [68]. Regarding the intestinal tract, transit time of digesta is approximately the same for goats and sheep; therefore, differences in susceptibility within this organ might include other factors such as protease types/concentrations within the intestinal lumen and/or cell-surface receptor densities on the mucosal epithelia [66]. Gross pathology of dose- and time-based experiments upon ligated ileal and colonic loops in goats and sheep show that the ileum is not affected by Etx; however, in the colon of either species the goblet cells are missing and there is an excess of mucus, leukocytes and sloughed epithelial cells into the lumen [69]. Colonic lesions are more severe in goats and the colonic loops of either species retain more water at two and four hours, *vs*. controls, post-Etx administration. There is also fluid volume change in toxin-treated ileal loops of goats, but minimally so in sheep (fluid evidenced only at four, but not two, hours following a maximum dose). Sodium efflux into the lumen may play a role in fluid accumulation within the ileum and colon, post-Etx exposure. 

### 4.1. Small Animal Models

The mode of action for Etx *in vivo* involves ion imbalance, endothelial disruption and edema. A vicious cycle is established by the toxin within the digestive tract and includes increased intestinal permeability that promotes higher, circulating levels of toxin throughout the body [61]. To more deeply understand Etx, or any other toxin, it is necessary to develop economically feasible, readily reproducible laboratory models *in vivo* that often include small animals such as mice. Furthermore, results from animal models critically advance vaccine and therapeutic developments. 

Various laboratories have published efforts involving different routes of murine intoxication by Etx. One laboratory reveals that an intravenous injection of Etx into mice (2–4 LD_50_) yields seizures within 60 minutes [70]. Excessive glutamate release is proposed to cause neuronal damage (cell death) targeting the hippocampus, particularly pyramidal cells. 

In mice injected intraperitoneally with sub-lethal/lethal doses of Etx, electron microscopy of brain reveals that within just 30 minutes the end-feet of astrocytes (cerebellum) begin swelling with evident damage to the capillary endothelium [71]. Cytoplasmic boundaries of Etx-affected astrocytes are not well defined and often ruptured. Neurons, relative to astrocytes, are not affected by the toxin in this model. Other noticeable changes elicited by Etx in the brain, over time, include: 1 h post-toxin–vacuole formation in endothelial cells; 2 h post-toxin–more prominent blebbing from endothelial cells and rupturing of astrocytes; 3 h post-toxin–morphological changes in the capillary endothelium that include multiple vacuoles with organelle disintegration, as well as a shrunken nucleus in granule cells; 5 h post-toxin–a few neurons become mildly shrunk with some vacuole formation; and 24 h post-toxin (following multiple sub-lethal injections)–platelet aggregation, swelling of astrocyte end-feet corresponding to clear, perivascular spaces. Perhaps hypoxia linked to capillary damage affects more drastically cell types with relatively low levels of oxidative enzymes, such as astrocytes and granule cells. This extensive study by Finnie also points out that varying results from similar studies involving Etx-induced damage to the brain could be linked to fixation techniques: immersion *vs*. perfusion [71]. 

One study with *C. perfringens* type D culture supernatants (late-log phase, *n =* 39 disease-causing strains collected from the 1940s–present), injected intravenously into mice, shows that Etx is required for lethality [72]. Trypsin added to these Etx-containing supernatants further increases lethal potency, while an Etx-specific monoclonal antibody prevents lethality. There is no correlation between alpha toxin and perfringolysin O concentrations with lethality in this model. For unknown reasons, not all supernatants of type D strains (approximately five percent) tested positive for Etx *in vitro*, yet by definition each of these bacteria contain the toxin gene. Such findings of silent Etx genes clearly have implications upon diagnosis and taxonomy. These studies nicely mimic those subsequently done in sheep, goats and mice, revealing that a genetic knockout of Etx derived from a wild-type D strain (sheep isolate, CN1020) does not cause disease following intraduodenal injection [73]. Complementation of this mutant with the wild-type toxin gene generates a phenotype that elicits Etx-based disease. In this study, clinical signs of Etx-intoxication in mice include depression, ataxia, circling and dyspnea. Overall, the above results strongly suggest that clostridial vaccines meant to prevent enterotoxemia should target Etx as an antigen [72,73]. 

An extensive study by Goldstein *et al*. shows that orogastric-administered Etx causes fluid accumulation in the murine ileum, with toxin binding preferentially throughout the villus, *vs*. crypt, surface [74]. Such a model is more cost-effective than large animal models (sheep and goats) for studying the intestinal/systemic effects of *C. perfringens* Etx. Interestingly, this same study reveals that Ussing chamber experiments of either mouse or rat ileum, incubated with Etx (8000 LD_50_ on mucosal surface for 60 minutes), does not disrupt transepithelial resistance. However, a similar experiment with basolateral application of toxin (30 LD_50_) shows damage within 40 minutes, likely disrupting tight junction gaps. Entry of toxin into the blood stream could facilitate this latter event. 

In this same study, horseradish peroxidase (44 kDa) minimally passes from the intestinal lumen into the zonula occludens of murine loops injected with Etx, suggesting minimal intestinal damage from the mucosal surface [74]. Intravenously-injected Evans blue dye, which binds to plasma proteins, leaks into the intestinal lumen within three hours after Etx administration in intestinal loops. Microscopy of rat and mouse intestinal loops reveals minimal effects upon the epithelium, with some shrunken cells and debris. Apoptotic cells are evident and contain condensed, fragmented nuclei plus degenerated organelles. Mild edema exists in the lamina propia of some, but not all, intestinal loops from animals treated with Etx. 

Another study by Fernandez-Miyakawa *et al*. uses oral administration of Etx-producing *C. perfringens* type D (*n =* 10 strains given at 8 × 10^9^ colony forming units (CFU)/mouse), leading to strain-dependent degrees of lethality (0%–100%) [75]. Seizures, hyper-excitability, depression, tubular necrosis of kidney and lung edema are signs of intoxication in these animals. Mice are passively protected by a monoclonal antibody against Etx, further suggesting a critical role of the latter in this model. However, there is no correlation between *in vitro* production (protein amounts) of Etx and lethality. 

### 4.2. Detection

The classic mouse assay involving toxin neutralization with *C. perfringens* type-specific antisera is used less these days, given non-animal alternatives. Relatively large numbers of *C. perfringens* type D (10^4^–10^7^ CFU/mL) can be isolated from Etx-affected sheep, but such data for goats are lacking to our knowledge [1]. Use of ELISA technology for specifically detecting Etx in intestinal contents is evidently, to date, one of the best ways to confirm intoxication [6,76]. As an example, ELISA testing (commercial kit) of various body fluids in Merino lambs (*n =* 15), each inoculated intraduodenally with *C. perfringens* type D culture (300 mL; 200–800 MLD_50_ of Etx), reveals respective 92%, 64% and 57% detection rates of Etx (0.075 MLD_50_/mL detection limit) in the ileum, duodenum and colon [6]. Animals were euthanized upon showing severe clinical signs (two to 26 hours post-toxin administration), and all fluids immediately frozen at −20 °C until processed within two months. Etx is also detected in 7% of the pericardial and aqueous humor fluids, but not in abdominal fluid or urine.

An extensive, ovine-based study by Uzal *et al*. assesses four techniques for detecting Etx (200–0.0075 MLD_50_/mL) spiked in intestinal contents, pericardial fluid and aqueous humor [76]. A capture ELISA format with intestinal contents, using polyclonal antibody (0.075 MLD_50_/mL detection limit) adsorbed to the plate well, is more sensitive than a monoclonal antibody capture ELISA (25 MLD_50_/mL), counter-immunoelectrophoresis (50 MLD_50_/mL) or toxin neutralization in mice (6 MLD_50_/mL). One major caveat with the polyclonal capture ELISA, counter-immunoelectrophoresis and mouse-lethal neutralization assays is that all use polyclonal antibodies against *C. perfringens* type D that could recognize antigens other than Etx. However, specificity of each assay is confirmed by using intestinal contents from sheep (*n =* 12) that died of causes other than Etx. Further studies using intestinal contents from goats with experimental or natural epsilon intoxication essentially confirm results from the spiked toxin experiments. Altogether, these results and those from other laboratories reveal that a properly crafted ELISA can be an effective method for detecting various toxins of *C. perfringens* [6,7,8,9]. 

A novel liquid chromatography–mass spectrometry technique uses immunoaffinity beads to initially concentrate Etx or protoxin from a complex matrix, such as serum or milk [10]. Detection limits of the assay are 5 ng/mL for either toxin or protoxin, and results are possible within four hours of sample processing. Mass spectrometry avoids cross-reactivity issues inherent in any antibody-based assay; however, ELISA and mass spectrometry do not determine whether the detected protein toxin is biologically active. 

Payne *et al*. discovered that a cell culture assay employing MDCK cells, purified Etx diluted in culture medium and specific neutralizing antibodies (monoclonal) against Etx correlates well with the mouse lethal assay [77]. Of the twelve different cell lines tested, the MDCK is the only one susceptible (~15 ng Etx/mL detection limit). The toxin-resistant cell lines are quite varied and include: (1) kidney (African green monkey, Vero and MA104; bovine, MDBK; rat, NRK-59F; porcine, LLC-PK1; feline, CRFK); (2) lung (rat, JTC-19); (3) neuronal (rat, B65); (4) B lymphocyte (human, B12); (5) intestinal epithelium (rat, IEC-6); and (6) monocyte/macrophage (murine, P388.D1). Readout involves inhibited metabolism of a viability stain (tetrazolium salt) by Etx-intoxicated cells, quantitated by spectrophotometry. Experiments are not reported using “dirty” field samples, such as intestinal contents, that can contain potential confounding factors. Various lines used in cell culture assays with various clostridial toxins, and neutralizing antibodies, have proven useful over time. 

In addition to established cell lines, freshly isolated human kidney cells (tubular epithelial) behave like MDCK cells following Etx, which includes blebbing and large complex formation [46,78]. Perhaps future *in vitro* studies that support discovery of therapeutics and vaccines for biodefense should use human, *vs*. canine, kidney cells? 

## 5. Management of Etx Intoxication: Therapy and Prophylaxis

Effective vaccines against Etx (described below) are readily available for animal use, thus obviating the need for a therapeutic in susceptible populations given this prophylaxis. In fact, vaccines that target *C. perfringens* type D (in particular Etx) are common reagents used for managing sheep and goat herds around the world [54]. There is certainly nothing (therapeutic or vaccine) against Etx approved for human use at this time. Findings from different laboratories and various *in vivo/in vitro* studies suggest that therapy is possible. Perhaps a proteomics-based approach following Etx exposure can reveal even more interesting, and unique, host-based targets for therapeutic intervention? This has recently been done using mice given Etx intravenously, with subsequent analysis of select organs (*i.e*., brain and kidney), plasma and urine for differentially expressed proteins [79]. The study reveals 136 different proteins with altered expression, post-toxin exposure. Similar to staphylococcal enterotoxins and expansion of distinct Vβ-bearing T cells [80], unique patterns of up- and/or down-regulated proteins might be useful in the future for diagnosing epsilon intoxication. 

A murine-based study using brain slices incubated with Etx and Etx-specific antibody shows that the toxin binds preferentially to the cerebellum, particularly oligodendrocytes and granule cells [81]. Current-clamp experiments with granule cells reveal that Etx decreases resistance, induces membrane depolarization and ultimately increases glutamate release. In addition to release of lactate dehydrogenase into the medium, incubation of primary-cultured granule cells with Etx causes membrane blebbing, rapid increase of intracellular calcium levels (within five minutes of toxin exposure) and glutamate release. Overall, these studies importantly provide a brain cell-specific target/assay *in vitro* for testing novel therapeutics. 

Related murine endeavors *in vivo* by Miyamoto *et al*. reveal that riluzole, a benzothiazole (234 Da) therapeutic for human amyotrophic lateral sclerosis that prevents presynaptic glutamate release, minimizes murine seizures as well as glutamate release induced by Etx [70,82]. These results occurred after an intraperitoneal dose of riluzole (16 mg/kg body weight) given 30 minutes before an intravenous dose (2 or 4 LD_50_) of Etx. However, the drug was evidently not used as a therapeutic (*i.e*., administered after toxin exposure). Experiments with rats also show that riluzole (8 mg/kg body weight), as well as glutamate antagonists MK-801 or CNQX (3 mg/kg body weight and 100 nmole, respectively), decrease hippocampus damage following intravenous injection of Etx (100 ng/kg body weight, a minimum lethal dose in rats) [70,82]. Etx-induced damage to the brain differs between rats and mice, as in rats the cortex and hippocampus (pyramidal cells) are most affected *vs.* the mouse cerebral cortex and granular layer of the cerebellum [82]. Overt effects of toxin in rats include upper body tremor, limb rigidity and muscular incoordination followed by hypotonus and paralysis. Perhaps varying susceptibility of brain regions between species may be linked to receptor densities, an aspect not clarified in subsequent literature to our knowledge.

A small-molecule library (151,616 compounds) and high-throughput screening have also been used with a MDCK cell-based assay (384-well plates) for discovering novel therapeutics against Etx [83]. In this study, Lewis *et al*. ultimately found three, structurally-unique inhibitors that afford protection against Etx but do not prevent toxin binding or oligomerization. These inhibitors seemingly affect pore function and/or an unidentified co-factor important in Etx intoxication. Two of these compounds (*N*-cycloalkylbenzamide and furo[2,3-*b*]quinoline) protect cells using various criteria. One experiment used a constant concentration of inhibitor added concomitantly with increasing concentrations of Etx needed to kill 50 percent of the cells (CT_50_). A post-exposure experiment shows that addition of either compound (50 μM) up to 10 minutes after toxin (25 nM) exposure is protective *in vitro*. Such results logically lead to efficacy studies in animals, yet to be done or at least published to our knowledge. Furthermore, these inhibitors are specific for Etx as the related *A. hydrophila* aerolysin is not inhibited in similar assays [83]. 

Another therapeutic approach against Etx includes dominant-negative inhibitors. These protein-based therapeutics have been used for other oligomer-forming bacterial toxins produced by Gram-positive and Gram-negative pathogens such as *Bacillus anthracis*, *Escherichia coli* and *Helicobacter pylori* [84,85,86]. Dominant-negative proteins are a recombinantly-attenuated version of a toxin generated by peptide deletion or amino acid substitution(s). Integration of a dominant-negative protein(s) into a wild-type toxin complex in solution or on a cell surface generates a non-functional toxin oligomer. Two dominant-negative inhibitors for Etx have been created via cysteine substitutions of isoleucine 51/alanine 114 or valine 56/phenylalanine 118 [87]. These particular paired mutations facilitate an intramolecular disulfide bond, restrict toxin insertion into the membrane and cause oligomer dysfunction (decreased heat/detergent stability plus poor pre-pore to pore transition) that ultimately inactivates Etx *in vitro*. Although used only as an Etx inhibitor *in vitro* (MDCK cells), dose-dependently effective at a 1, 2, 4 or 8 (wild-type toxin):1 (dominant negative) mole mixture, these unique constructs should perhaps be tested *in vivo* for therapeutic and vaccine potential. 

Additional studies in mice show that the epsilon protoxin delays time to death when given intravenously before activated toxin. Protection presumably occurs by competitive occupation of cell-surface receptors, namely in the brain, by the protoxin [56]. These data further suggest the feasibility of a receptor-targeted approach for prophylaxis/therapy. In fact, Buxton had discovered many years prior to these studies that a formalin toxoid of the protoxin protects mice up to 100 minutes after Etx exposure [88]. These collective results indeed make sense as the protoxin and toxin share the same binding site and dissociation constant (K_d_ ~ 4–6 nM) on MDCK cells [89]. Plasma membrane integrity plus an unidentified O-linked glycoprotein are important for toxin binding, as determined by detergent solubilization, pronase or lectin pretreatment of cells. The detergent type (Triton X-100, sodium deoxycholate or sodium cholate), concentration and temperature also affect toxin binding. The latter results suggest cryptic receptors naturally hidden by membrane protein(s), lipid(s) and/or carbohydrate(s). This same study shows that Etx targets the distal and collecting tubules, but not those proximal, in cryosectioned mouse kidneys [89]. To date, work with receptor antagonists other than the epsilon protoxin have evidently not been pursued (at least published) by various laboratories.

Identifying a toxin’s cell-surface receptor and understanding how toxin molecularly interacts with it can be very useful in formulating receptor-based therapies. The latter include toxin-binding antagonists and therapeutic targeting of susceptible cell populations throughout the body. Early receptor-binding studies employing radio-iodinated Etx reveal a heat-labile sialoglycoprotein, as pretreatment of rat synaptosome membranes with heat (70–80 °C for 10 minutes), neuraminidase, lipase or pronase effectively reduces saturable binding of the toxin [90]. The K_d_ values of Etx binding to rat brain homogenates and synaptosomal membrane fractions are 2.5 and 3.3 nM, respectively, thus very similar to K_d_ values in kidney cells [89,90]. Furthermore, a snake-venom presynaptic neurotoxin (beta-bungarotoxin from the many-banded krait, *Bungarus multicinctus*) that blocks acetylcholine release dose-dependently decreases binding of Etx, suggesting a common receptor for these protein toxins from diverse organisms [90]. However, the presynaptic neurotoxin produced by *C. botulinum* type A had no effect upon binding of Etx. Furthermore, sialidase pre-treatment of kidney cells and synaptosomes suggests different receptors for Etx [48,90]. 

Unique studies involving gene-trap mutagenesis show that hepatitis A virus cellular receptor 1 (HAVCR1) is a receptor, or co-receptor, for Etx in MDCK cells [91,92]. It is also possible that HAVCR1 promotes Etx-induced: (1) intracellular signaling due to toxin binding or increased ion flow; and/or (2) protein-protein interactions (*i.e*., homo-oligomer formation). There are eight other proteins, including sphingomyelin synthase 2, that when not expressed lead to varying cell resistance towards Etx [91]. These results suggest that multiple cell factors play a role during epsilon intoxication; however, HAVCR1 is the only protein consistently linked to increased expression and Etx susceptibility in other cell lines. 

The natural role of HAVCR1 (also known as KIM-1, Kidney Injury Molecule-1) involves T-regulatory cells and maintaining immunological balance throughout the body. HAVCR1 is a class I, integral-membrane, O-linked glycoprotein containing multiple isoforms varying within a mucin-like domain containing multiple glycosylation sites. The 100, but not 90, kDa variant of HAVCR1 binds Etx, perhaps due to increased length of the extracellular, mucin-like domain containing approximately 57 glycosylation sites [91]. Domain I tyrosines (residues 29, 30, 36, 196) on Etx are surface-accessible and contribute to toxin binding to HAVCR1 [34,92]. Replacement of any of these tyrosines with glutamic acid yields a non-toxic protein unable to bind cells (MDCK), yet these molecules possess a similar circular dichroism (CD) spectrum and resistance to trypsin digestion as the wild-type toxin. In contrast, mutagenesis of tyrosines 16 and 20, or phenylalanine 37 does not decrease epsilon cytotoxicity. Replacement of phenylalanine 199 with glutamic acid yields a non-toxic protein with a unique CD spectrum, suggesting conformational differences *vs.* wild-type toxin. Although recent studies with Etx and HAVCR1 are intriguing, further studies must be done to more clearly understand the intimate interactions between these molecules.

A subsequent, and contrasting, study by Bokori-Brown *et al*. interestingly suggests that aforementioned tyrosines 29, 30, 36 and 196 (when individually changed to alanine) do not play a role in binding/cytotoxicity of ACHN cells, in contrast to MDCK cells [92,93]. Such findings might suggest an alternative receptor(s) exploited by Etx, perhaps mediated by a beta-octyl-glucoside binding site within domain III. It is also quite possible that replacement of these tyrosines with an electroneutral alanine, *vs*. an electronegative glutamic acid, is too subtle of a change. Cumulative data from different laboratories indeed make this aspect of understanding epsilon intoxication complex and clearly unresolved to date. 

As stated earlier, Etx is primarily of veterinary concern and vaccines are used in the field [94,95,96]. Before the use of commercial vaccines against *C. perfringens* type D (Etx), more lambs died from enterotoxemia than all other diseases combined [94]. Lambs from ewes vaccinated three to four weeks before parturition have higher passive antibody titers against Etx *vs.* lambs from ewes vaccinated six weeks before parturition. Ewe-derived antibodies can evidently protect lambs up to twelve weeks of age. Recommendations that lambs be vaccinated twice before six weeks of age, regardless of ewe vaccination status, are evidently not warranted [94]. From a cost/benefit perspective, lambs marketed by five months of age may not need vaccinations if ewes are appropriately vaccinated against clostridial enterotoxemia [94]. 

Parenteral hyperimmune sera can also generate passive protection for three to four weeks in weaned lambs [97]. However, animals showing clinical signs of epsilon intoxication cannot be saved by anti-toxin. Colostrum (bovine) containing anti-Etx antibodies, following multiple immunizations with a clostridial multicomponent vaccine, can also provide protection (circulating antibody) against Etx when fed to lambs within 48 hours of birth [98]. This antibody–rich product can be particularly useful for weak and/or orphaned lambs, as well as stored in relatively large quantities for long periods at −20 °C. It is also possible that a monoclonal antibody targeting a critical epitope, like the membrane insertion region of Etx, could be a better characterized, purified therapeutic *vs*. a heterogeneous population of antibodies in serum or colostrum [99,100]. Antibodies generated by either active or passive immunization can effectively thwart *C. perfringens* Etx, when timely present. 

Human and animal vaccines have been quite effective over time against various diseases. However, many veterinary vaccines, such as those against *C. perfringens* (or other bacterial pathogens) and associated toxins, are frequently formaldehyde toxoids of culture filtrates that may also contain whole cells [101]. Although these vaccines can be efficacious and relatively inexpensive, they are typically too crude for human use [102]. Current veterinary vaccines containing epsilon toxoid can vary in protective efficacy [95]. This latter study tested seven commercially available, epsilon-toxoids available in Brazil and remarkably found only two of sufficient potency. Clearly, quality control can vary greatly which ultimately affects the bottom line: protection against disease. 

The vaccination schedule to counter epsilon enterotoxemia varies too, depending upon the animal species [96]. Two doses, containing aluminum hydroxide adjuvant, are usually given two to six weeks apart and then followed by an annual (sheep) or quarterly (goat) boost. Evidently immunoreactivity (antibody titers and duration) towards Etx-based vaccine is less in goats *vs.* sheep. An attempt to remedy this problem involved a specific study in goats, comparing liposome- *vs.* aluminum hydroxide- based vaccines (three doses at three week intervals) of epsilon toxoid [96]. The latter proved much more superior. In fact, the liposome-based vaccine did not elicit a significant antibody response towards epsilon toxoid. In animals, and likely humans too, an efficacious vaccine against Etx will probably not be a “one and done” that generates lasting protection. 

In contrast to relatively crude (multiple antigen) vaccines for Etx, recombinant technology can be helpful in many ways towards a better vaccine. The gene for Etx was first successfully cloned, sequenced and expressed in 1992, making subsequent work possible [103]. As one example, recombinant *E. coli* expressing Etx that is then inactivated by 0.5% formaldehyde at room temperature for 10 days can evidently be used as a cost-effective vaccine [95]. This vaccine can be administered at a much lower protein concentration (0.2 mg/dose) than current, chemically-detoxified epsilon toxoids derived from crude cultures of *C. perfringens* [95]. The standard recommendation by the National Institute for Biological Standards and Control (United Kingdom) is 9.2 mg of native Etx per dose. This latter study used a 1:1 emulsion of toxoid (0.2 mg/dose):aluminum hydroxide (2.5%–3.5%) for vaccination of goats, sheep and cattle [95]. Use of a more purified vaccine also affords easier quality control. A human vaccine against Etx will likely involve chemically (*i.e*., formaldehyde) or recombinantly (*i.e*., mutation of critical residues needed for receptor binding and/or oligomerization) detoxified versions of purified protein. 

In regards to recombinant-based attenuation, replacement of just one amino acid (histidine 106) with proline results in a non-toxic form of Etx (H106P) when tested by MDCK cytotoxicity and mouse lethality [104]. In contrast, a serine or alanine replacement of histidine 106 does not eliminate toxicity. The H106P protein (0.27 nmole/mouse given intraperitoneally with Freund’s incomplete adjuvant) affords vaccine-based protection against 1000 LD_50_ of intravenous, wild-type toxin. 

Further vaccine refinement, with minimal risk of aforementioned toxicity, involves use of a linear, B-cell epitope (amino acids 40–62 of Etx) linked to a carrier molecule (*E. coli* heat-labile toxin B subunit) [105]. In theory this could be useful, but a small protein fragment may adopt an unnatural conformation (*vs.* that found on native protein) and subsequently elicit antibodies that don’t readily bind native protein. This particular study did not determine if this construct elicits protective antibodies in an animal model for epsilon intoxication. In our opinion, replacement of select amino acids involved in a critical step during the intoxication process (*i.e*., cell binding, oligomerization, *etc*.) typically does not grossly alter native conformation and thus represents a better vaccine strategy offering multiple epitopes to the immune system. 

## 6. Conclusions

*C. perfringens* is one of the most “toxic” bacteria known, using different protein toxins in different ways to perpetuate itself. Many of these toxins are commonly linked to various diseases in many mammalian species. In particular, the Etx has been studied by various groups and is primarily a veterinary concern for some large animals. Vaccines of varying quality are available to combat epsilon enterotoxemia, for veterinary use only. A human equivalent for biodefense is not commercially available. As a therapeutic or prophylactic for humans, toxin-specific immunoglobulins might be helpful following nefarious application of Etx. Clearly, there is much more to learn regarding Etx, how it works and how to protect against it. Such knowledge goes a long way towards bettering the lives of animals and humans.
